# Cisplatin sensitivity of testis tumour cells is due to deficiency in interstrand-crosslink repair and low ERCC1-XPF expression

**DOI:** 10.1186/1476-4598-9-248

**Published:** 2010-09-16

**Authors:** Svetlana Usanova, Andrea Piée-Staffa, Ulrike Sied, Jürgen Thomale, Astrid Schneider, Bernd Kaina, Beate Köberle

**Affiliations:** 1 Institute of Toxicology, Clinical Centre of University of Mainz, Obere Zahlbacher Strasse 67, 55131 Mainz, Germany; 2 Institute of Medical Biostatistics, Epidemiology and Informatics, Clinical Centre of University of Mainz, Germany, Obere Zahlbacher Strasse 69, 55131 Mainz, Germany; 3 Institute for Cell Biology, University of Duisburg-Essen Medical School, Hufeland Strasse 55, 45122 Essen, Germany

## Abstract

**Background:**

Cisplatin based chemotherapy cures over 80% of metastatic testicular germ cell tumours (TGCT). In contrast, almost all other solid cancers in adults are incurable once they have spread beyond the primary site. Cell lines derived from TGCTs are hypersensitive to cisplatin reflecting the clinical response. Earlier findings suggested that a reduced repair capacity might contribute to the cisplatin hypersensitivity of testis tumour cells (TTC), but the critical DNA damage has not been defined. This study was aimed at investigating the formation and repair of intrastrand and interstrand crosslinks (ICLs) induced by cisplatin in TTC and their contribution to TTC hypersensitivity.

**Results:**

We observed that repair of intrastrand crosslinks is similar in cisplatin sensitive TTC and resistant bladder cancer cells, whereas repair of ICLs was significantly reduced in TTC. γH2AX formation, which serves as a marker of DNA breaks formed in response to ICLs, persisted in cisplatin-treated TTC and correlated with sustained phosphorylation of Chk2 and enhanced PARP-1 cleavage. Expression of the nucleotide excision repair factor ERCC1-XPF, which is implicated in the processing of ICLs, is reduced in TTC. To analyse the causal role of ERCC1-XPF for ICL repair and cisplatin sensitivity, we over-expressed ERCC1-XPF in TTC by transient transfection. Over-expression increased ICL repair and rendered TTC more resistant to cisplatin, which suggests that ERCC1-XPF is rate-limiting for repair of ICLs resulting in the observed cisplatin hypersensitivity of TTC.

**Conclusion:**

Our data indicate for the first time that the exceptional sensitivity of TTC and, therefore, very likely the curability of TGCT rests on their limited ICL repair due to low level of expression of ERCC1-XPF.

## Background

Over 80% of patients with metastatic testicular germ cell tumours (TGCT) can be cured using cisplatin-based chemotherapy [1]. Since introduction of cisplatin in the clinic it became a component of standard treatment of ovarian, cervical, head and neck, lung and bladder cancer. Unfortunately, however, none of these malignancies can be treated with a similar efficiency as TGCT [2]. Understanding why TGCT are sensitive to chemotherapeutic drugs is likely to have implications for the improved treatment of other types of cancer. Cell lines derived from TGCT retain their exceptional sensitivity to many chemotherapeutic drugs, reflecting the clinical response [3]. Using testis tumour cell lines as a model system may help to define the molecular basis for this hypersensitivity [4].

The major DNA lesions induced by cisplatin are intrastrand DNA crosslinks between two guanines or guanine and adenine, accounting together for ~90% of the platination lesions. In contrast, interstrand crosslinks (ICLs) between the two DNA strands are minor lesions, accounting for less than 5% of all cisplatin lesions [5]. Intrastrand crosslinks are repaired by nucleotide excision repair (NER), whereas ICLs are removed by ICL repair, a process less well understood than NER [6]. A survey of repair proteins revealed that the expression level of the ERCC1-XPF endonuclease, which is involved in repair of both intrastrand crosslinks and ICLs, is low in testis tumour cell lines compared to other tumour lines [7] suggesting that ERCC1-XPF might contribute to the observed cisplatin sensitivity.

Previously, we showed that testis tumour cells (TTC) remove DNA platination damage more slowly from the whole genome and from single genes than cisplatin resistant tumour cells indicating a deficiency in the repair of DNA platination [8]. Here we extended this study and investigated whether TTC are impaired in the repair of ICLs, which have not been studied before in TTC. As a model system we used TTC and bladder cancer cells as proven examples of cisplatin sensitive and resistant cell lines, respectively [8]. We determined the expression level and over-expressed ERCC1-XPF in TTC and down-regulated the repair proteins in bladder cancer cells. The data revealed for the first time that the exceptional sensitivity of TGCT to cisplatin is associated with a low capacity for repairing ICLs, and that levels of ERCC1-XPF are rate-limiting. This is clinically important as it demonstrates that ERCC1-XPF could be used as a target to enhance the response of tumours to ICL-inducing drugs.

## Results

### Removal of GpG-intrastrand crosslinks in TTC

**Figure 1:**
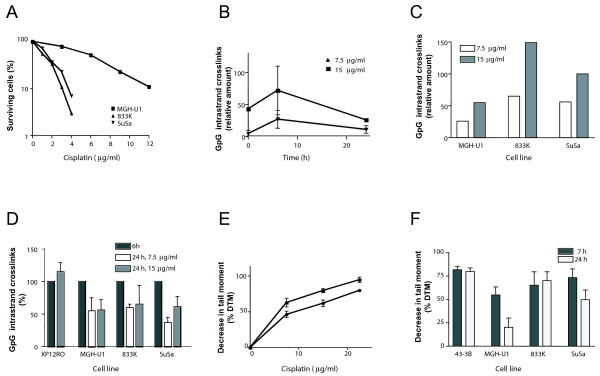
**Induction and removal of cisplatin-induced DNA lesions**. A) Clonogenic cell survival curves of 833 K and SuSa testis tumour cells and MGH-U1 bladder cancer cells after cisplatin treatment for 1 h. B) MGH-U1 bladder cancer cells were treated with cisplatin (7.5 or 15 μg/ml) for 1 h, DNA was isolated 0 h, 6 h or 24 h post-treatment and examined for the presence of GpG-intrastrand crosslinks. C) MGH-U1 bladder cancer cells, 833 K and SuSa testis tumour cells were treated with cisplatin (7.5 or 15 μg/ml) for 1 h. The levels of GpG-intrastrand crosslink were determined 6 h post-treatment. D) MGH-U1 bladder, 833 K and SuSa testis and XPA-deficient XP12RO cells were treated with cisplatin (7.5 or 15 μg/ml) for 1 h, GpG-intrastrand crosslink levels were determined 6 and 24 h post-treatment. The levels measured 6 h post-treatment were set to 100%. The levels measured 24 h post-treatment were corrected for dilution due to DNA synthesis during the recovery period. Each point represents the mean of 3 to 6 replicate experiments. E) MGH-U1 bladder (circles) and 833 K testis (squares) tumour cells were treated with cisplatin for 1 h and analysed for ICL cross-linking 7 h post-treatment. The results are the means of three independent experiments. F) MGH-U1 bladder, 833 K and SuSa testis and ERCC1-deficient 43-3B cells were treated with cisplatin (15 μg/ml) for 1 h, the level of ICL cross-linking was determined 7 and 24 h post-treatment. Each column represents the mean of 3 to 6 replicate experiments.

To investigate cisplatin-damage repair in TTC, we used the cell lines 833 K and SuSa and compared them with MGH-U1 bladder cancer cells. Earlier studies using colony formation assays revealed a 3-fold higher cisplatin sensitivity of 833 K and SuSa cells (for comparison of sensitivity of TTC with bladder cancer cells see Figure 1A) suggesting these lines represent a useful model system for cisplatin sensitive and resistant cells, respectively [8]. DNA intrastrand crosslinks are the major DNA lesions induced by cisplatin. To investigate whether repair of cisplatin-induced intrastrand crosslinks is impaired in TTC, experiments were performed to measure their removal from genomic DNA. GpG-intrastrand crosslinks were detected using a lesion-specific antibody [9]. Time-response curves using MGH-U1 cells showed that GpG-intrastrand crosslinks were formed directly after cisplatin treatment, and at a higher level 6 h later (Figure 1B). This is in line with previous findings showing that following a 1 h cisplatin treatment the level of intrastrand crosslinks peaked after 4-6 h [10]. For repair experiments we therefore treated the cells with cisplatin for 1 h and determined the level of GpG crosslinks 6 h later (for the maximum level of GpG adducts) and 24 h later for determining their repair. The amount of GpG-intrastrand crosslinks measured 6 h post-treatment was set to 100%. The GpG-intrastrand crosslink levels measured 24 h post-treatment were corrected for dilution due to DNA synthesis during the recovery period to rule out a reduction of GpG damage because of DNA replication. Quantification revealed that the initial amount of GpG intrastrand crosslinks was about 50% lower in bladder cancer cells compared to TTC (Figure 1C). Post-treatment removal of GpG-intrastrand crosslinks in cells treated with cisplatin in shown in Figure 1D. No reduction in the level of GpG-intrastrand crosslinks was observed in XP12RO cells, which are deficient in the NER protein XPA and were therefore included as a control for repair deficiency. In MGH-U1 bladder cancer cells the amount of GpG-intrastrand crosslinks was significantly reduced by 45% after 24 h. This reduction was similar to that seen in TTC where the amount of GpG-intrastrand crosslinks was reduced by about 35% after 24 h. Since a repair capacity of cisplatin-induced intrastrand crosslinks in the range of 40-50% indicates repair proficiency [10], the data indicate that TTC are able to repair cisplatin-induced intrastrand adducts.

### Induction and repair of ICLs in testis tumour cells

Induction and repair of cisplatin-induced ICLs was investigated using a modification of the comet assay, which permits detection of ICLs at the single cell level [10]. Cisplatin-induced ICLs peak between 7-9 h post-treatment (data not shown). Therefore, cells were treated with increasing concentrations of cisplatin for 1 h and the amount of ICLs was determined 7 h later. The level of ICLs increased in a concentration dependent manner (Figure 1E). To measure the repair of ICLs, cells were treated with cisplatin (15 μg/ml, 1 h) and the amount of ICLs was determined 7 and 24 h after treatment. As a control we used the ERCC1-deficient cell line 43-3B [11]. The level of ICLs did not change in 43-3B cells, which is in line with the observation that these cells are deficient in ICL repair. For MGH-U1 cells the ICL level was reduced by about 50% after 24 h suggesting proficiency of repair of cisplatin-induced ICLs in the bladder cancer cells (Figure 1F). No reduction in the ICL level after 24 h was observed in 833 K cells, and a small reduction in ICL level was observed in SuSa cells indicating impaired ICL repair. Statistical analysis confirmed the difference in ICL repair capacity between MGH-U1 and 833 K (p ≤ 0.001) and MGH-U1 and SuSa (p ≤ 0.001). Taken together, the data revealed that TTC are impaired in the repair of cisplatin-induced ICLs.

### Persistence of DNA damage in testis tumour cells

**Figure 2:**
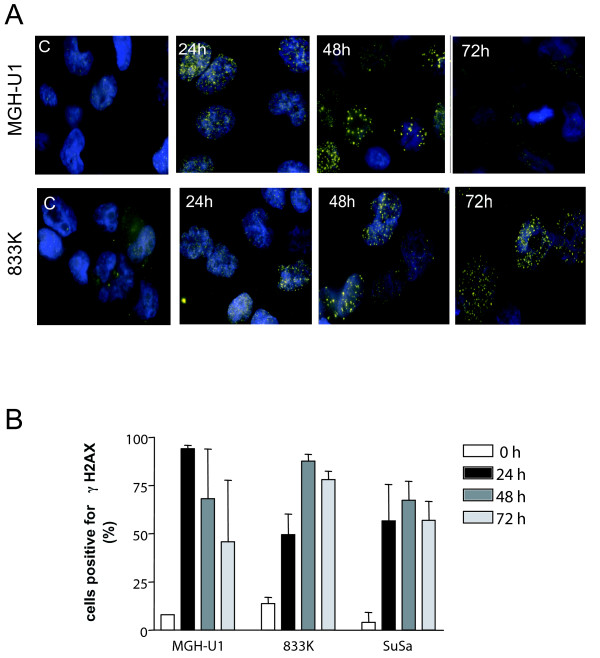
**γH2AX staining in bladder and testis tumour cell lines**. A) Representative examples for MGH-U1 and 833 K cells treated with cisplatin (6 μg/ml) for 1 h and stained for γH2AX at the indicated time points. C: untreated control. DNA counterstaining is with DAPI. B) Quantification of γH2AX staining of MGH-U1 bladder, 833 K and SuSa testis tumour cells after exposure to cisplatin (6 μg/ml) for 1 h, followed by a recovery period of 24, 48 or 72 h. For each data point, more than 400 nuclei/experiment were examined, the γH2AX fluorescence was determined and the % cells with a fluorescence intensity more than 500 units (control value) were plotted. Each column represents the mean of 3 replicate experiments.

γH2AX formation can be used as a marker for DNA damage (notably DNA double-strand breaks) associated with ICLs [12]. We therefore treated the cells with cisplatin for 1 h and stained for γH2AX 24, 48 and 72 h post-treatment. Representative examples of γH2AX staining for MGH-U1 bladder and 833 K testis tumour cells are shown in Figure 2A. γH2AX immunofluorescence was measured in more than 400 cells per time point. The results were expressed as % of γH2AX positive cells, which were defined as having a fluorescence signal above 500 units (Figure 2B). Over 90% of MGH-U1 cells stained positive for γH2AX when analyzed 24 h after cisplatin treatment, and the percentage of positive cells was decreased 48 and 72 h post-treatment. In contrast, γH2AX formation after cisplatin treatment persisted in 833 K and SuSa cells (Figure 2B). It has been suggested that following the induction of interstrand crosslinks the persistence of γH2AX formation may result from defective processing of ICLs [12]. The data, therefore, support the conclusion of an impaired repair of ICLs in TTC.

### Chk1 and Chk2 phosphorylation by cisplatin treatment

**Figure 3:**
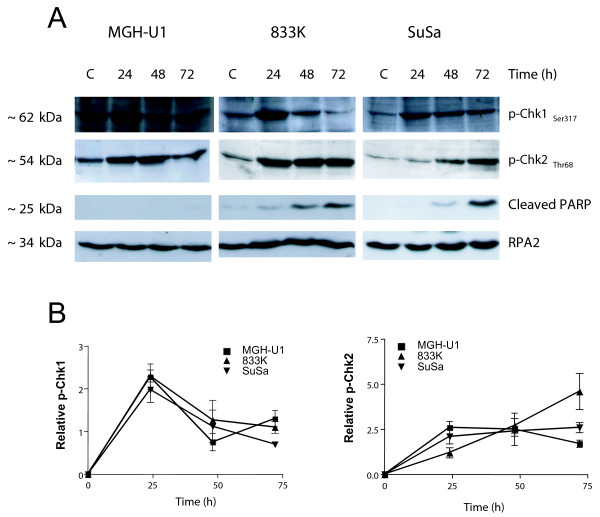
**Phosphorylation of Chk1 and Chk2 and PARP-1 cleavage in testis versus bladder cancer cell lines**. A) Immunoblot analysis of p-Chk1_Ser317_, p-Chk2_Thr68 _and cleaved PARP-1 in 50 μg protein extract of MGH-U1 bladder, 833 K and SuSa testis tumour cells. RPA2 was used as a loading control. Cells were harvested at the indicated time points after treatment with cisplatin (6 μg/ml) for 1 h. B) Quantification of the data obtained in immunoblots.

The exact mechanism of how initial cisplatin lesions are recognized and the signal becomes transmitted to the apoptotic machinery is still not entirely clear [13]. However, both Chk1 and Chk2 have been implicated in cisplatin damage signaling [14,15]. We therefore investigated the phosphorylation of Chk1 and Chk2 at different times following cisplatin treatment. PARP-1 cleavage was also investigated as a hallmark of apoptosis. Representative examples of immunoblots are shown in Figure 3A, demonstrating that cisplatin induces activation of Chk1_Ser317 _and Chk2_Thr68 _as well as PARP-1 cleavage. PARP-1 cleavage was clearly more pronounced in TTC lines than in MGH-U1 (Figure 3A), which is in line with the higher apoptotic response in these cells following cisplatin. Chk1 was transiently induced with a peak level in all lines at 24 h after the onset of cisplatin treatment. For Chk2 a marked difference was observed 3 d after treatment where the activation occurred at higher level in TTC lines than in MGH-U1 cells (Figure 3A and 3B). This was taken to indicate that checkpoint activation occurs as a sustained response in TTC, presumably because of the presence of non-repaired DNA lesions.

### Effect of over-expression of ERCC1-XPF on ICL repair and cisplatin sensitivity in testis tumor cells

**Figure 4:**
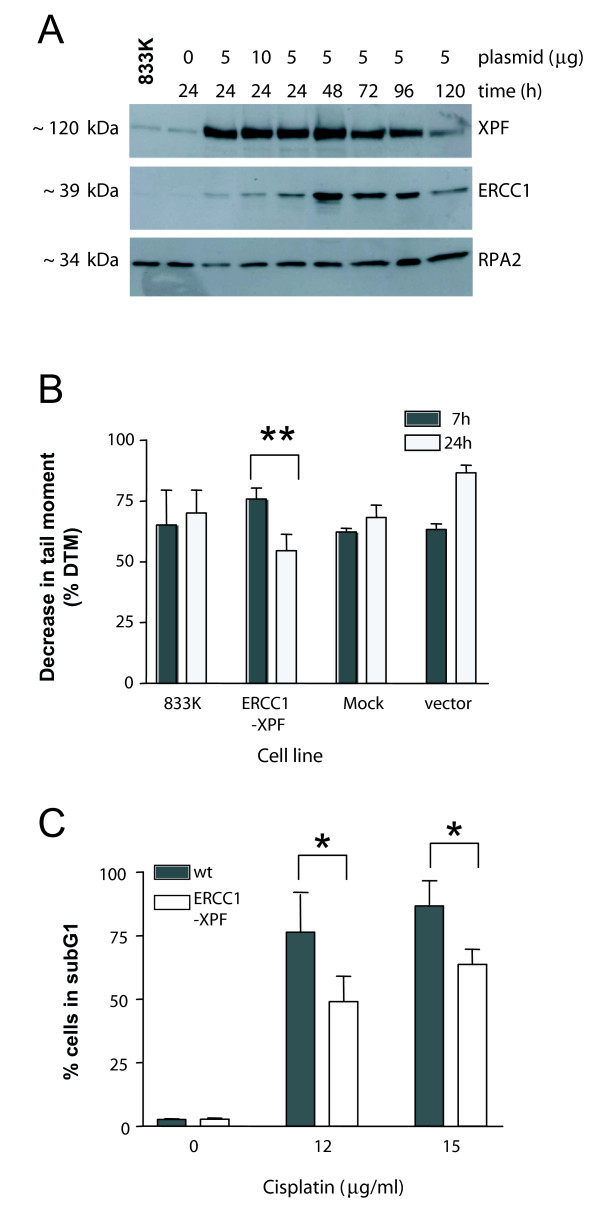
**Effect of ERCC1-XPF over-expression on ICL repair and cisplatin sensitivity**. A) 833 K cells were transfected with plasmid pEF6(XPF-IRES-ERCC1). Immunoblot analysis of XPF and ERCC1 proteins in extracts harvested at the indicated time points. RPA2 served as loading control. B) 833 K cells (non-transfected, transfected with pEF6(ERCC1-IRES-XPF), transfected with vector pEF6, mock transfected) were treated with cisplatin (15 μg/ml) for 1 h, the level of interstrand crosslinking was determined 7 and 24 h post-treatment. Each column represents the mean of 3 to 6 replicate experiments. C) Cells were transfected with plasmid pEF6(XPF-IRES-ERCC1) for 24 h, treated with cisplatin (12 and 15 μg/ml) for 1 h and post-incubated for 96 h. Apoptosis was measured by flow cytometry (sub-G_1 _content). * represents statistical significance.

The endonuclease ERCC1-XPF is implicated in the repair of cisplatin-induced ICLs [16]. As the level of ERCC1-XPF is low in TTC [7] we hypothesized that ERCC1-XPF is the rate-limiting factor responsible for the observed impaired ICL repair in TTC. 833 K cells were transiently transfected with the bi-cistronic mammalian expression vector pEF6(XPF-IRES-ERCC1) and the levels of ERCC1 and XPF proteins were investigated. Immunoblot analysis showed an increase in both ERCC1 and XPF proteins over time (Figure 4A). Over-expression of ERCC1-XPF did not increase the level of ICL repair in MGH-U1 (data not shown). However, over-expression of ERCC1-XPF in 833 K cells resulted in significant repair of cisplatin-induced ICLs (Figure 4B). When 833 K cells were transfected without DNA (mock) or with a vector control (vector) we observed no repair of ICLs (Figure 4B). Statistical analysis confirmed the difference in ICL repair between 833 K untransfected cells and ERCC1-XPF over-expressing cells (p ≤ 0.001). Altogether these data suggest that over-expression of ERCC1-XPF abrogated the ICL repair deficiency in TTC.

To investigate whether the increased ICL repair has an impact on cellular sensitivity towards cisplatin, ERCC1-XPF was over-expressed in 833 K cells before treatment with cisplatin. The cells were treated with cisplatin (12 and 15 μg/ml, 1 h) and apoptosis was determined 96 h post-treatment. Over-expression of ERCC1-XPF clearly reduced the frequency of cells in subG1 indicating that ERCC1-XPF had a protective effect on apoptosis induction by cisplatin (Figure 4C). These findings suggest that low levels of ERCC1-XPF contribute to cisplatin sensitivity in TTC. We should note that the decrease in sensitivity was not dramatic, most likely because of a low transfection efficiency usually observed with TTC which are difficult to transfect because of their extreme sensitivity towards most experimental manipulations.

### Effect of ERCC1-XPF down-regulation on cisplatin sensitivity in MGH-U1 bladder cancer cells

**Figure 5:**
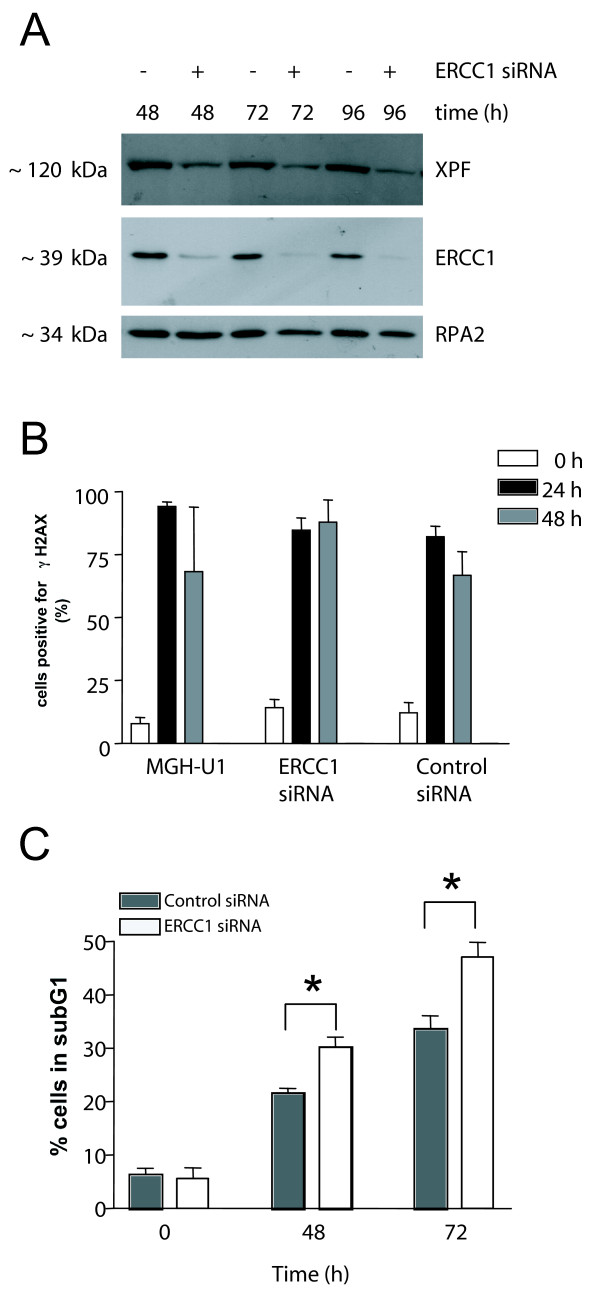
**Effect of ERCC1-XPF down-regulation on cisplatin ICL damage and sensitivity**. A) MGH-U1 cells were transfected with 10 nM ERCC1 siRNA and analysed 48, 72 and 96 h after transfection for ERCC1 and XPF expression. RPA2 served as loading control. B) MGH-U1 cells were transfected with 10 nM ERCC1 siRNA or control siRNA, cultivated for 96 h, treated with cisplatin (6 μg/ml) for 1 h and post-incubated for 24 or 48 h. γH2AX fluorescence was quantified in MGH-U1 parental cells and cells transfected with either ERCC1 siRNA or control siRNA. For each data point, more than 400 nuclei/experiment were examined, the γH2AX fluorescence was determined and the % cells with a fluorescence intensity more than 500 units (control value) were plotted. Each column represents the mean of 3 independent experiments. C) MGH-U1 cells were transfected with 10 nM ERCC1 siRNA or control siRNA, cultivated for 96 h, treated with cisplatin (9 μg/ml) for 1 h and post-incubated for 48 or 72 h. Apoptosis was measured by flow cytometry (sub-G_1 _content). * represents statistical significance.

As a proof of principle for demonstrating a contribution of ERCC1-XPF for cisplatin sensitivity, we down-regulated ERCC1-XPF in MGH-U1 cells using siRNA against ERCC1. ERCC1 and XPF form a tight complex [17]. The proteins are unstable and are rapidly degraded without its partner [18]. It is therefore possible to reduce both ERCC1 and XPF protein using siRNA against ERCC1. We transiently transfected MGH-U1 cells with ERCC1 siRNA and investigated ERCC1 and XPF levels by immunoblotting. A strong decrease of both ERCC1 and XPF protein levels was observed (Figure 5A). To investigate the effect of down-regulation of ERCC1-XPF on cisplatin-induced ICL damage, cells were transfected with ERCC1 siRNA, treated with cisplatin for 1 h and investigated for gH2AX formation as a marker for damage related to cisplatin ICLs 24 and 48 h later (Figure 5B). Between 80 and 90% of cells stained positive for γH2AX when analyzed 24 h after cisplatin treatment. In MGH-U1 parental cells and cells transfected with control siRNA the percentage of positive cells was decreased 48 h post-treatment. In contrast, γH2AX formation after cisplatin treatment persisted in MGH-U1 cells transfected with ERCC1 siRNA (Figure 5B). These data indicate that down-regulation of ERCC1-XPF lead to impaired processing of ICLs in bladder cancer cells. To further investigate the effect of down-regulation of ERCC1-XPF on cisplatin sensitivity, cells were transfected with ERCC1 siRNA, treated with cisplatin, and apoptosis was determined 48 and 72 h later. Down-regulation of ERCC1-XPF increased the sensitivity to cisplatin, as shown by the higher level of apoptosis (Figure 5C). The increase in sensitivity was statistically significant but small, perhaps due to the relatively long cultivation period following after siRNA transfection, and variations in transfection efficiency. The data support the view that ERCC1-XPF is a key factor in determining cisplatin sensitivity of TTC.

## Discussion

In this study, we demonstrate for the first time that TTC are impaired in the repair of ICLs, which are minor lesions formed in response to cisplatin. The repair defect accounts, at least in part, for the sensitivity towards cisplatin. We also identified ERCC1-XPF as a factor underlying the impaired ICL repair. The observation of impaired ICL repair in TTC is supported by sustained cisplatin-induced γH2AX formation, which was paralleled by a late and sustained activation of Chk2 and late PARP-1 cleavage. The importance of ERCC1-XPF in processing ICLs has also been demonstrated in ERCC1-deficient mouse and hamster cell lines. In ERCC1 deficient MEFs persisting γH2AX foci were shown after treatment with the ICL-inducing agent MMC [16]. Similar data were obtained for cisplatin in ERCC1 mutated UV96 hamster cells [12]. In renal cells cisplatin lead to sustained activation of Chk2, which in turn resulted in activation of the apoptotic pathway [14]. We found sustained activation of Chk2 following cisplatin treatment in TTC and conclude that persisting ICLs result in DSB formation that lead to a long-term DNA damage response and finally activation of the apoptotic pathway [19].

In contrast to the deficiency in ICL repair, TTC were proficient in removing intrastrand crosslinks. Cisplatin-induced intrastrand crosslinks are removed by NER, and our findings suggest that testis tumour cells are basically NER proficient. This is supported by the finding that 833 K cells are capable of repairing UV-induced photoproducts, which are removed exclusively by NER [20]. These findings in living cells, however, are in contrast to the low NER capacity, which was observed in experiments using cell-free extracts of TTC lines including 833 K [21]. TTC lines have low levels of the NER proteins XPA and ERCC1-XPF [7,21], and we hypothesize that low levels of these proteins together with the short incubation times applied are apparently inadequate to sustain efficient NER *in vitro *assays while they are apparently sufficient for performing NER in living cells. The findings reported here also contrast with earlier studies where it was shown that TTC remove DNA platination damage more slowly from the whole genome and from single genes compared to bladder cancer cells [8]. The discrepancy might be explained by considering that in these earlier experiments removal of total platination was investigated, which is a quite crude measure of DNA damage, while here the repair of GpG-intrastrand adducts was studied using a highly sensitive immuno-assay. In addition, in the experimental set-up used earlier platination levels were measured directly after cisplatin treatment and compared to the level 24 h later, while here we compared the level of cisplatin-induced intrastrand adducts 6 and 24 h post-treatment because intrastrand adduct formation peaks at 6 h post-treatment [10]. One could argue that the efficiency to remove cisplatin-induced mono-adducts is reduced in TTC while the resulting intrastrand crosslinks are recognized by the NER system because they cause more distortion of the DNA structure. This suggestion is supported by the observation that GpG-intrastrand crosslink levels were higher in TTC compared to bladder cancer cells. This, however, is unlikely to cause the increased sensitivity of TTCs since earlier studies showed that some bladder cancer cells exhibit up to 3 times the initial platination level compared to TTC, but still were considerably more resistant to the drug [8].

In contrast to NER, the ICL repair pathway is not well understood and a number of models for ICL repair have been discussed [6]. *In vitro *and *in vivo *data implicate the NER factor ERCC1-XPF in the repair of ICLs in addition to its role in NER [16,17,22]. We tested the hypothesis that low levels of ERCC1-XPF are responsible for the impaired ICL repair in TTC. Over-expression of ERCC1-XPF resulted in ICL repair in 833 K cells suggesting that low levels of ERCC1-XPF contribute to impaired ICL repair, while the reduced levels of ERCC1-XPF are still sufficient to perform NER in TTC. It is not yet known at which level ERCC1-XPF becomes a rate-limiting factor for NER. For the NER factor XPA we found that levels of this protein had to be reduced to less than 10% of that of normal to render XPA rate-limiting for NER [23]. It has been shown that the transient participation time of XPA in a single NER event is 4 to 6 min [24], a similar dynamic behaviour was demonstrated for ERCC1-XPF [25]. Possibly, even low levels of XPA and ERCC1-XPF are sufficient due to the short time of a single NER event, while this might not be the case for the more complex ICL repair process.

A number of studies have implicated repair deficiency as a reason for cellular sensitivity towards cisplatin. We found that over-expression of ERCC1-XPF protein increased the resistance of 833 K cells to cisplatin. The effect was not dramatic most likely due to the fact that 833 K cells are sensitive to experimental manipulations such as transfection. Nevertheless the data show that ERCC1-XPF mediated ICL repair has a protective effect on TTC and indicate that low ERCC1-XPF levels contribute to cisplatin sensitivity in these cells. In support of this we showed that down-regulation of ERCC1-XPF rendered MGH-U1 bladder cancer cells more sensitive to cisplatin. In line with this hypothesis is the finding that acquired resistance towards cisplatin is often correlated with an increased expression of ERCC1 [26,27]. In clinical studies high ERCC1 expression was associated with resistance to platinum containing therapy in various human cancers including colorectal cancer, ovarian cancer or NSCLC [28-31]. Altogether the clinical studies together with *in vitro *data suggest that ERCC1 may serve as a reliable predictive marker for resistance to cisplatin in human cancers.

The observation that the clinically relevant sensitivity of TTC is, at the level of DNA, due to impaired ICL repair, raises the interesting question about inhibition of ICL repair as a strategy to increase the efficacy of chemotherapy. In general, inhibition of DNA repair has the potential to enhance the cytotoxicity of anticancer agents. Preclinical studies have confirmed that modulation of repair pathways such as base excision repair, strand break repair, MGMT and PARP can enhance the sensitivity to DNA damaging agents [32]. For a number of repair inhibitors clinical studies are now under way [33-35]. In order to inhibit the repair of ICLs different approaches can be envisaged, with ERCC1 as a potential key anticancer target. As ERCC1 has no known catalytic activity, ERCC1-XPF or ERCC1-XPA protein-protein interactions might be a target for sensitization strategies. UCN-01, which reduces the ERCC1-XPA interaction, has been shown to increase cisplatin toxicity [36]. However, enzymatic activities have proven to be more successful targets than disruption of protein-protein interactions. Therefore, targeting the endonuclease activity of XPF directly might also be a successful approach. Thus, our findings on TTC may encourage the search for strategies aimed at sensitizing other cancers to cisplatin-based chemotherapy by inhibiting ICL repair.

## Conclusion

Our study identified ICLs as critical cytotoxic lesions induced by cisplatin in TTC, which are not repaired because of a low level of expression of ERCC1-XPF. Therefore, the repair complex ERCC1-XPF appears to be responsible at DNA level for the exquisite cisplatin sensitivity of testis tumors. Overexpression of ERCC1-XPF resulted in repair of cisplatin induced ICLs and decreased cisplatin sensitivity suggesting that persisting ICLs in TTC trigger DSB formation (as demonstrated by gammaH2AX) that in turn activates the apoptotic pathway. Our study suggests that targeting ERCC1-XPF might be a strategy for improving the therapeutic efficacy of cisplatin for other types of cancer.

## Materials and methods

### Cell culture and cisplatin treatment

833 K and SuSa human TGCT cell lines, MGH-U1 bladder carcinoma cells and XPA-deficient XP12RO were described previously [7,23]. 43-3B is an ERCC1-deficient CHO hamster cell line [11]. All cell lines were grown in RPMI 1640 medium supplemented with 10% heat-inactivated fetal calf serum, L-glutamine (PAA) and 5% antibiotics (penicilline/streptavidine). For drug treatment cells were incubated with cisplatin for 1 h at 37°C in a humidified atmosphere.

### Determination of apoptosis

Apoptosis was measured by flow cytometry (sub-G_1 _content). After treatment with cisplatin and postincubation in fresh medium, cells were harvested, fixed with ethanol (70%) and stained with propidium iodide (17 μg/ml) after RNase (30 μg/ml) digestion. Samples were analyzed on a FACSCalibur (Becton Dickinson, Germany). Accumulated data were analyzed using WinMDI Software.

### Colony formation assays

Colony formation assays were performed according to [8].

### Construction of mammalian expression vector

ERCC1 and XPF cDNAs were cut from plasmid pET30B(+)ERCC41 [37]. XPF cDNA between restriction sites XbaI and Not I was ligated into the mammalian expression vector pEF6 (Invitrogen) digested with SpeI and NotI. ERCC1 cDNA between XbaI restriction sites was ligated into pEF6(XPF) digested with XbaI. IRES sequence was amplified by PCR using primers with 5'ends complementary for the NotI restriction site. The PCR product was digested with NotI and ligated into pEF6(XPF-ERCC1). The resulting vector pEF6(XPF-IRES-ERCC1) was used for transfection studies.

### Transfection experiments

To over-express ERCC1-XPF transient transfections were performed. Cells were incubated for 24 h with medium containing Effectene transfection reagent (Qiagen) and 2 μg of vector pEF6(XPF-IRES-ERCC1) or 2 μg of vector pEF6 containing no insert. After transfection the cells were washed and treated with cisplatin for 1 h. Transient knock-down of ERCC1 was achieved by transient transfection of 10 nM ERCC1 siRNA (Dharmacon RNA technologies: D-006311-02). siRNA was delivered using Dharmacon siRNA transfection reagent according to the manufacturer's instructions. A non-targeting siRNA was used in control experiments and was purchased from Qiagen (AllStars Negative Control siRNA).

### Detection of GpG-intrastrand crosslinks with lesion specific antibody

Cells were treated with cisplatin for 1 h and harvested immediately or incubated in fresh medium for another 6 or 24 h. DNA was isolated using the Master-Pure™ Complete DNA and RNA Purification kit (Epicentre^® ^Biotechnologies, USA). For each time point 4 μg DNA was used in duplicate for detection of cisplatin-induced GpG adducts. DNA was denatured (95°C for 10 min) and placed on ice immediately. Ice-cold ammonium acetate was added (final concentration 1 M), DNA was applied to a nylon Hybond-N+ membrane (Amersham) using a slot blot apparatus (Hybridot Manifold, Bethesda Research Laboratories). The membrane was washed with ammonium acetate (1 M), incubated in 5 × SSC for 5 min and washed in water. The DNA was fixed onto the membrane by baking at 80°C for 2 h. The membrane was blotted in PBS/0,2% Tween-100, 5% non-fat dry milk for 2 h, incubated with an antibody specific for GpG-intrastrand crosslinks [9] at a dilution of 1/500 at 4°C overnight. Peroxidase-conjugated secondary anti-rat antibody (1/2,000) was used for 2 h in blocking buffer. GpG-intrastrand crosslinks were visualized by chemiluminescence. SynGene software was used for quantification. The percentage of lesions remaining at 24 h was calculated in comparison to the lesions present 6 h post-treatment. To assess DNA synthesis during the recovery period, cells were labeled with 50 nCi/ml [^14^C]thymidine for 24 h prior to cisplatin treatment. Dilution factors (specific activity of DNA at time point/specific activity of DNA at 0 h) were determined for the 24 h repair period.

### Determination of cisplatin-induced interstrand crosslinking, statistical analysis

The detection of interstrand crosslinking was investigated using a modification of single cell gel electrophoresis (comet assay) as described previously [10]. Exponentially growing cells were treated with cisplatin for 1 h, harvested after 7 h and 24 h and diluted to a density of 2.5 × 10^4 ^cells/ml. All cisplatin-treated samples and one control were subjected to 8 Gy X-irradiation to induce random strand breakage, one unirradiated control was also included. The cells were lysed and subjected to electrophoresis. The presence of ICLs retards migration of the irradiated DNA during electrophoresis, resulting in reduced tail moment compared to control cells. To prevent repair of DNA breaks after irradiation, cells were kept on ice. Immediately after irradiation the cells were embedded in 0.5% low melting point agarose on microscope slides which were pre-coated with with 0,5% low melting point agarose. After the agarose solidified, the slides were placed in cold lysis buffer (2.5 M NaCl, 100 mM EDTA, 10 mM Tris, 1% Na-Laurylsarcosinate, pH 10) and incubated for 1 h at 4°C, followed by incubation in alkaline electrophoresis buffer (300 mM NaOH, 1 mM EDTA, pH > 13) for 30 min to denature DNA. Electrophoresis was carried out for 15 min at 25 V in the dark. Following electrophoresis the slides were incubated in neutralization solution (0.4 M Tris pH7.5) 3 × for 5 min, rinsed with H_2_O and fixed with absolute ethanol for 5 min. Slides were allowed to dry overnight, stained with propidium iodide (50 μg/ml), and comets were analyzed using a Nikon MIKROPHOT FXA fluorescence microscope. Fifty cells per slide were analyzed using Komet 4.0.2 Assay Software (Kinetic Imaging Ltd, Liverpool). The value of the tail moment was used to describe the rate of migration of DNA out of the nucleus during electrophoresis. The tail moment is calculated as product of percentage of DNA in the comet tail and distance between the head and tail. The presence of ICLs retards migration of the irradiated DNA during electrophoresis, resulting in a reduced tail moment compared to the untreated control. The amount of ICLs was therefore determined by comparing the tail moment of the irradiated cisplatin-treated samples with irradiated untreated samples and unirradiated untreated controls. The level of interstrand crosslinking is proportional to the decrease in tail moment (DTM) in the irradiated drug treated sample compared to the irradiated untreated control. The % DTM was calculated using the following formula described by [10]: % DTM = [1 - (TM_D IR _- TM_C U_)/(TM_C IR _- T_C U_)] × 100, where TM_D IR _is the mean tail moment of the cisplatin treated irradiated sample, TM_C IR _is the mean tail moment of the irradiated control sample and TM_C U _is the mean tail moment of the unirradiated control sample. Statistical analysis using the software program SPSS (SPSS Inc., USA) was performed using % DTM of 150-200 cells. Kruskal-Wallis one-way analysis of variance by ranks was used to compare whether ICL repair capacity differed within the group of four cell lines. To compare two independent groups of sampled data Mann-Whitney U test was used.

### γH2AX immunocytochemistry

Cells were plated at a density of 3 × 10^5 ^per dish on cover slips in 60 mm dishes and incubated for 24 h at 37°C. The cells were treated with cisplatin (6 μg/ml) for 1 h and incubated in fresh medium for 24, 48 and 72 h, respectively. Cells were quickly rinsed with PBS and then fixed with 4% formaldehyde for 20 min at RT, followed by ice-cold methanol absolute at -20°C for up to 72 h. The fixed cells were blocked with 5% BSA in PBS/0.3% Triton-X100 for 1 h at RT and then incubated with a 1:1,000 dilution of anti-phospho H2AX_Ser139 _antibody (Millipore) over night at 4°C. After washing 3 times in PBS the samples were incubated with a 1:500 dilution of a Alexa-fluor 488 conjugated goat anti-mouse antibody (Invitrogen) for 1-2 h at RT in the dark, followed by staining with DAPI (100 ng/ml) for 30 min. 10 μl mounting medium was added, and the cover slips containing the cells were mounted onto microscope slides. Fluorescence images were captured using a Zeiss microscope (ImageM1 AX10) and analysed using Metafer software (MetaSystems).

### Immunoblotting

Protein extraction and SDS gel electrophoresis were perfomed according to [7]. The antibodies used were: XPF 1/5,000 dilution of polyclonal antibody RA1 [21]; ERCC1 1/1,500 dilution of polyclonal antibody RWO18 [17]; RPA2 1/5,000 dilution of monoclonal antibody 9H8 (NeoMarkers); phospho-Chk1 1/1,000 dilution of polyclonal antibody (Bethyl Laboratories); phospho-Chk2 1/1,000 dilution of polyclonal antibody (Epitomics); PARP-1 1/1,000 dilution of monoclonal antibody raised against amino acids 22-219 (BD); anti-rabbit IgG 1/2,000 dilution (DAKO) or anti-mouse IgG 1/5,000 dilution (DAKO). RPA2, which is a housekeeping protein, was used as loading control since our previous data showed relatively little variation in RPA2 levels for different cancer cell lines [7].

## List of abbreviations

TGCT: testicular germ cell tumours; TTC: testis tumour cells; ICL: interstrand crosslink; NER: nucleotide excision repair.

## Competing interests

The authors declare that they have no competing interests.

## Authors' contributions

SU, US and AP-S participated in the design of the study and carried out the cellular and molecular analyses. AS performed the statistical analysis. JT participated in the analysis of repair studies. BK participated in the analysis of the experiments and worked on the manuscript. BKö conceived of the study, participated in it's design and drafted the manuscript. All authors have read and approved the final manuscript.
